# The differentiation of mesenchymal bone marrow stem cells into nerve cells induced by *Chromolaena odorata* extracts

**DOI:** 10.12688/f1000research.108741.1

**Published:** 2022-03-01

**Authors:** Kartini Eriani, Desriani Desriani, Suhartono Suhartono, Miftahul Jannah Br Sibarani, Ichsan Ichsan, Dedy Syafrizal, Hadhymulya Asmara

**Affiliations:** 1 Department of Biology, Faculty of Mathematics and Natural Sciences, Universitas Syiah Kuala, Banda Aceh, Aceh, 23111, Indonesia; 2 Research Center for Biotechnology, National Research and Innovation Agency, Cibinong, Bogor, West Java, 16911, Indonesia; 3 Faculty of Medicine, Universitas Syiah Kuala, Banda Aceh, Aceh, 23111, Indonesia; 4 Hotchkiss Brain Institute, University of Calgary, Calgary, AB, T2N 4N1, Canada

**Keywords:** Chromolaena odorata, mesenchymal stem cell, nerve cell, cell differentiation

## Abstract

**Background:** Mesenchymal stem cells (MSCs) can differentiate into nerve cells with an induction from chemical compounds in medium culture. *Chromolaena odorata* contains active compounds, such as alkaloids and flavonoids, that can initiate the transformation of MSCs into nerve cells. The aim of this study was to determine the potential of methanol extracted *C. odorata* leaf to induce the differentiation of bone marrow MSCs into nerve cells.

**Methods:** A serial concentration of *C. odorata* leaf extract (0.7–1.0 mg/mL) with two replications was used. The parameters measured were the number of differentiated MSCs into nerve cells (statistically analyzed using ANOVA) and cell confirmation using reverse transcription polymerase chain reaction (RT-PCR).

**Results:** The results showed that the *C. odorata* extract had a significant effect on the number MSCs differentiating into nerve cells ( *p *< 0.05) on the doses of 0.8 mg/ml with 22.6%. Molecular assay with RT-PCR confirmed the presence of the nerve cell gene in all of the samples.

**Conclusions: **In conclusion, this study showed the potential application of *C. odorata* leaf extract in stem cell therapy for patients experiencing neurodegeneration by inducing the differentiation of MSCs into nerve cells.

## Introduction

Stem cells have received significant attention in the medical field because of their extended growth characteristics and ability to differentiate into other cell types. Neural stem cells (NSCs) can repair damaged brain cells by undergoing differentiation into nerve cells and glial cells. ^1^ In adults, NSCs are found in the subventricular zone (SVZ) of the lateral ventricles and the sub-granular zone (SGZ) of the hippocampus. In the SVZ, the differentiation of NSCs into nerve cells occurs through migration along the rostral migration stream to the olfactory bulb. While in the SGZ, NSCs migrate to the granule cell layer. ^2^ However, the capacity of NSCs to replace lost cells is limited and the regeneration of nerve cells in the mammalian brain, despite spontaneous regeneration, does not compensate for all lost nerve cells. ^3^ As an alternative to NSCs, mesenchymal stem cells (MSCs) could be employed to repair damaged nerve cells. MSCs can differentiate into various mesenchymal cells comprising fibroblasts, chondrocytes, osteoblasts, myoblasts, and adipocytes. ^4^

Bone marrow derived MSCs become the most potent stem cells for cell replacement because of their efficiency, high proliferative capacity, and immunological naivety. A suitable media is required as a habitat for nerve growth for the successful differentiation of MSCs into nerve cells. The media can be modified by adding fibroblast growth factor-2 (FGF-2), FGF-8, brain-derived neurotrophic factor (BDNF), or particular substrates. ^5^ However, there are some challenges in the differentiation process of MSCs because of the low resistance of nerve cells during the culture process. To overcome this, an inducer is needed during the MSC differentiation process. Genetics, epigenetic, and chemical inducers have been used. ^6^^,^^7^ Recently, researchers have utilized plant extracts to increase cell production by enhancing proliferation and protecting stem cells during the growing phase. ^8^

*Chromolaena odorata* has pharmacological efficiency in wound healing by involving cell proliferation, inhibiting cell apoptosis, and contracting the collagen lattice. ^9^ The plant extract contains flavonoids and alkaloids, which contribute to establishing proper growth of nerve cells, protecting the extracellular environment in the nervous system, inducing glial cell secretion of nerve growth factors, and protecting neurons from oxidative stress-induced apoptosis. ^10^ In this study, we investigated the potential of *C. odorata* leaf extract as an inducer for MSC differentiation from bone marrow into nerve cells, where its expression was then detected using reverse transcription polymerase chain reaction (RT-PCR).

## Methods

### Study design

An *in vitro* experiment study was conducted to assess the potential of *C. odorata* leaf extract as an inducer for MSC differentiation into nerve cells. The MSCs were isolated from the bone marrow of a *Mus musculus* (the house mouse) and four concentrations of *C. odorata* extract were tested. Randomization and blinding of the animals were not required in this study. Two control groups (positive and negative) were used. The effect of *C. odorata* extract on MSC differentiation into nerve cells were measured after nine days of incubation.

### Chromolaena odorata extraction

Extraction was conducted using a maceration method with methanol as the solvent. First, *C. odorata* leaves were washed using water and sun-dried for three days. Dried leaves were then crushed into simplicial powder using a DF-15 grinder (CGOLDENWALL). Then 500 grams of simplicial powder were mixed with 1 liter of 96% methanol for five days, filtered and stored for two days at room temperature. The filtrate was evaporated using a BUCHI R-300 Rotary Evaporator (BÜCHI Labortechnik AG, Meierseggstrasse, Postfach, Germany) at 40°C with 80–90 rpm before the extract was kept in a container.

### Preparation of Dulbecco’s modified eagle’s medium (mDMEM)

A stock culture medium of 1000 ml mDMEM was prepared by mixing 1 g of DMEM powder with aquadest and was homogenized. Then 0.37 g of NaHCO _3_, 100 μL of non-essential amino acids (Sigma-Aldrich Pte Ltd, Singapore), 100 μL of insulin transferrin selenium (Thermo Fisher Scientific, Carlsbad, CA, US), 100 μl of gentamicin (Sigma-Aldrich Pte Ltd, Singapore), and 10 mL of 10% fetal calf serum (Sigma-Aldrich Pte Ltd, Singapore) were added to the solution. The mixture was then sterilized using microfiltration with a diameter of 0.22 μm. ^11^

### Housing and husbandry of animals

The male *M. musculus* mice of the strain BALB/c (eight weeks of age) were acclimated and fed *ad libitum* with standardized feed for one week under laboratory conditions with a 12 h light-dark cycle, 60% of humidity with 23 ^o^C. To minimize the number of animals, six mice were used with assumption that the number of MSCs from one mouse was enough for one set of repetitions (i.e., with four repetitions in this study, two other mice were prepared for any unexpected event such as if the MSCs did not grow).

The animal facility was cleaned and disinfected regularly. During the acclimation the animal facility was kept quiet with controlled environmental conditions. If there were abnormal behaviors (apathy or increased aggression), the animals were excluded from the study and returned to the breeding center of the Faculty of Veterinary Medicine, Universitas Syiah Kuala. The mice were anesthetized with 0.01 mL of ketamine (Troy Laboratories PTY Limited) and 0.01 mL of xylazine (Troy Laboratories PTY Limited) and the animals were sacrificed using a cervical dislocation technique per protocol. ^12^ It was ensured that all animals were dead before any autopsy was conducted. The autopsies were conducted by a certificated veterinarian.

### Mesenchymal stem cells extraction

The femur and tibia were removed from the mice and washed using FBS solution to remove muscle and fat tissue. Both ends of the bone were cut, then the marrow contents were removed using a syringe and stored in a culture dish containing FBS and NBCS. The bone marrow suspension was pipetted and centrifuged at 3,000 rpm for 10 min and then rinsed four times with FBS and once with mDMEM to remove as many single cells as possible. ^10^ Before incubation, the number of cells (cells/mL) was counted using a hemocytometer.

### Mesenchymal stem cells exposure with *C. odorata* extract

A 1 mL of bone marrow suspension containing 1 × 106 cells was placed in a petri dish containing mDMEM medium and cultured in an incubator humidified with 5% CO _2_ at 37°C. After one day of incubation, the media was replaced and 10 μL of plant extract was added with a concentration of 0.7 mg/mL (group M1), 0.8 mg/mL (M2), 0.9 mg/mL (M3), and 1.0 mg/mL (M4) as treatments group and no extract was added as the negative control. The cells were incubated for nine days with media replacement every two days. ^11^

### Evaluation of differentiated bone marrow mesenchymal stem cells

*Enumeration of differentiation nerve cells*

Cells were observed under a CKX41 inverted microscope (Olympus Life Science, Tokyo, Japan) with 100x magnification in 16 fields of view. The enumeration was performed in three replications and the results averaged.

*Nerve cell gene confirmation using reverse transcription polymerase chain reaction (RT-PCR)*

The gene of nerve-like cells was detected using RT-PCR. The RNA was extracted using Z6011 ReliaPrep RNA Cell Miniprep System following the manufacturers’ protocol (Promega, Madison, WI, USA). Then 1 μL RNA was converted into a reverse transcription template cDNA using GoScript Reverse Transcriptase and random primer (Promega, Madison, WI, USA). The reaction condition was maintained at 25°C for 5 min, 37°C for 60 min, and 70°C for 15 min. The amplification of the cDNA was carried out using GoTaq qPCR Master Mix (Promega, Madison, WI, USA) and 2 μL forward and reverse primers (β-actin and β-tubulin 3). The detailed primer sequences are presented in Table 1. ^13^ The PCR product was then analyzed on 1% agarose gel electrophoresis with TAE buffer run at 80 V for 60 min then visualized using UV light at 312 nm.

**Table 1.** Primers for DNA amplification.

Primer	Forward and reverse sequence
β-actin	F: 5’ ATGAAGATCCTGACCGAGCG 3’
	R: 5’ TACTTGCGCTCAGGAGGAGC 3’
β-tubulin	F: 5’ TTCCCCCGCTTGCATTT 3’
	R: 5’ TGCCCCGGGCTGTTAGT 3’

### Statistical analysis

To compare the differentiation of the nerve cells among different doses of *C. odorata*, the data were analyzed using analysis of variance (ANOVA) followed by Duncan’s post hoc test with a significance level of 5%. All analyses were conducted using SPSS software version 20 (IBM SPSS, Chicago, IL, USA) (RRID:SCR_019096).

### Ethics statement

Ethical clearance was obtained from the Research Ethics Committee of the Faculty of Veterinary Medicine, Universitas Syiah Kuala (No 110/KEPH/VI/2021) - PT Bimana Indomedical (No.R.07-20-IR). All efforts were made to ameliorate any suffering of animals. Efforts were made to minimize the pain, suffering and distress experienced by the research animals. The animals were provided with appropriate housing with *ad libitum* feeding, the appropriate anesthesia was used to minimize pain before the animals were sacrificed and all procedures were conducted by a certificated veterinarian with the animal care training.

## Results and discussion

### Number of differentiated mesenchymal stem cells into nerve like cells

**Figure 1.**
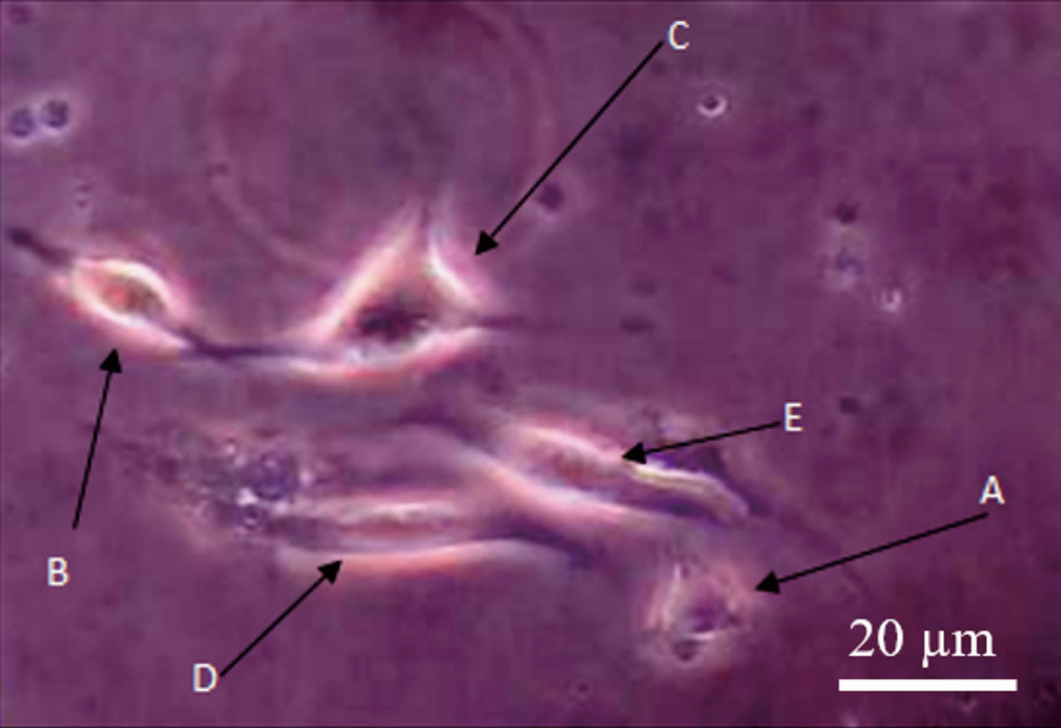
Nerve cells observed in medium culture: (A) Apolar; (B) Bipolar; (C) Multipolar; (D and E) undifferentiated.

The leaf extract of *C. odorata* with a concentration of 0.8 mg/mL was the most optimum concentration for inducing cell growth with mean total cells of 147.67. Therefore, the morphology examination of differentiated MSCs into nerve-like cells was conducted using this optimal dose. The observations focused on differentiated nerve-like cells with cytoplasmic appendages. The cell's cytoplasm consisted of dendrites and axons, which indicated the cells were similar to nerve cells. Herein, it was observed that there were some variations of the nerve-like cells, such as nerve cells (neuroblast) with apolar ( Figure 1A), bipolar ( Figure 1B), and multipolar ( Figure 1C) forms. Some undifferentiated MSCs ( Figure 1D and E) were also observed.

The differentiation process of nerve-like cells in medium culture showed the development steps of nerve-like cell formation, followed by dendrite extension and enlargement of the cytoplasm and dendritic bodies. Apolar nerve-like cells ( Figure 1A) are the first development step of nerve cells, indicated by round and undeveloped extension of dendrites. The nerve-like cells then developed into bipolar form with two dendritic appendages ( Figure 1B). The bipolar nerve-like cells then differentiated into multipolar forms characterized by multi dendritic extensions ( Figure 1C). ^14^ Bipolar nerve-like cells are classified as young nerve-like cells and multipolar as mature nerve-like cells. ^15^

**Figure 2.**
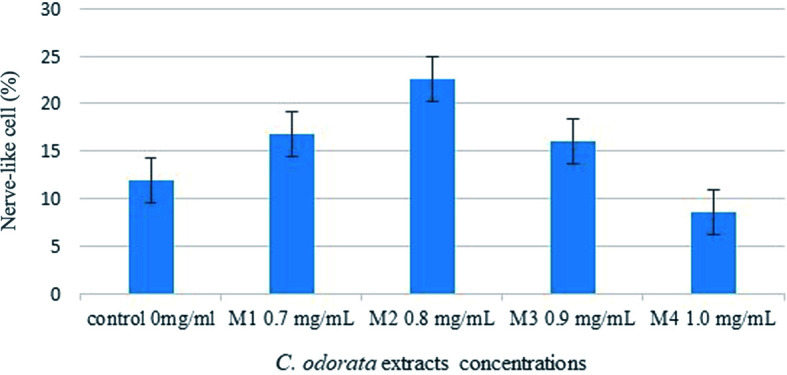
Differentiated nerve-like cells in different concentrations of *C. odorata* extract.

The mean number of nerve-like cells showed that the doses of *C. odorata* extracts had a significant implication on the differentiation ( Table 2 and Figure 2). The M2 group (0.8 mg/mL) had a higher number of differentiated nerve-like cells compared to other treatments (i.e., M1 (0.7 mg/mL), M3 (0.9 mg/mL), and M4 (1.0 mg/mL) and control groups. This suggested that 0.8 mg/mL was the most optimum concentration for inducing the differentiation of MSCs into nerve-like cells.

**Table 2.** The total number of nerve-like-cells in DMEM medium with different doses of methanol extract of *C. odorata.*

Treatments	Cell numbers (Mean ± SD)	Mean nerve-like cells (Mean ± SD)	Nerve-like cells (%)
Negative control	86.00 ± 08.84	10.25 ± 02.98	11.9
M1 0.7 mg/mL	112.88 ± 15.7	19.00 ± 01.82 *	16.8
M2 0.8 mg/mL	147.67 ± 88.1	33.25 ± 0.957 *	22.6
M3 0.9 mg/mL	88.57 ± 13.6	14.25 ± 03.52 *	16.0
M4 1.0 mg/mL	102.48 ± 33.6	08.85 ± 02.64	8.6

* Significant at p < 0.05 compared to the negative control group.

Based on the statistical analysis, there was no significant difference between the control and M4 (1.0 mg/ml) group on inducing differentiated MSC into nerve cells. This indicated that 1.0 mg/mL had an inhibition effect on the cell culture. The decrease in the number of differentiated cells may be due to the use of excessive concentration. Extracts with excessive concentrations can cause cytotoxic effects on cells, so that cell growth becomes inhibited. ^16^ Based on these results, the induction by *C. odorata* leaf extract on the differentiation of MSCs into nerve cells was better carried out at concentrations ranging from 0.7 to 0.9 mg/ml.

The leaf extract of *C. odorata* used in this study has active chemical compounds, such as flavonoids and alkaloids. Flavonoids are compounds that may also trigger nerve cell formation (neurodegeneration) by increasing the production of nitric oxide (NO). NO is used as a potent activator of the soluble guanyl cyclase enzyme. This enzyme is responsible for forming cyclic-guanosine monophosphate (cGMP). ^17^ The concentration of cGMP can also decrease the conversion of cGMP into GMP by adding phosphodiesterase-5 (PDE-5), an inhibitor enzyme. ^18^ The presence of NO might assist the conversion of cGMP into sildenafil. Sildenafil can lead to the increased protein expression of phosphatidylinositol 3-kinase (PI3K)-Akt. PI3K-Akt is one of the essential regulation factors for the neurodegeneration process and transmission of information to neuronal progenitor cells. ^19^ Moreover, cGMP activates cGMP-phosphokinase G (PKG) pathways leading to the increasing cycle-adenosine monophosphate (cAMP) and element-binding protein (CREB) response that is essential for neuroblast viability. ^20^ BDNF, cGMP, and P13K activity use Wnt signaling, which is the primary pathway in the differentiation process by increasing the number of receptors. ^21^^,^^22^

A study found that alkaloids had an essential role in leading to neuroprotective effects by the inhibition of oxidative stress and the up regulation of BDNF expression. ^23^ BDNF is one of the factors that can trigger the expression of nerve cells, which is essential in regulating plasticity, immune, and nerve formation (neuro-regeneration). ^24^ BDNF can affect the survival and development of nerve cells by activating kinase receptor B enzyme in nerve cells and ganglia cells. ^25^

### Detection of nerve cell gene (β-tubulin 3 and β-actin) with RT-PCR

**Figure 3.**
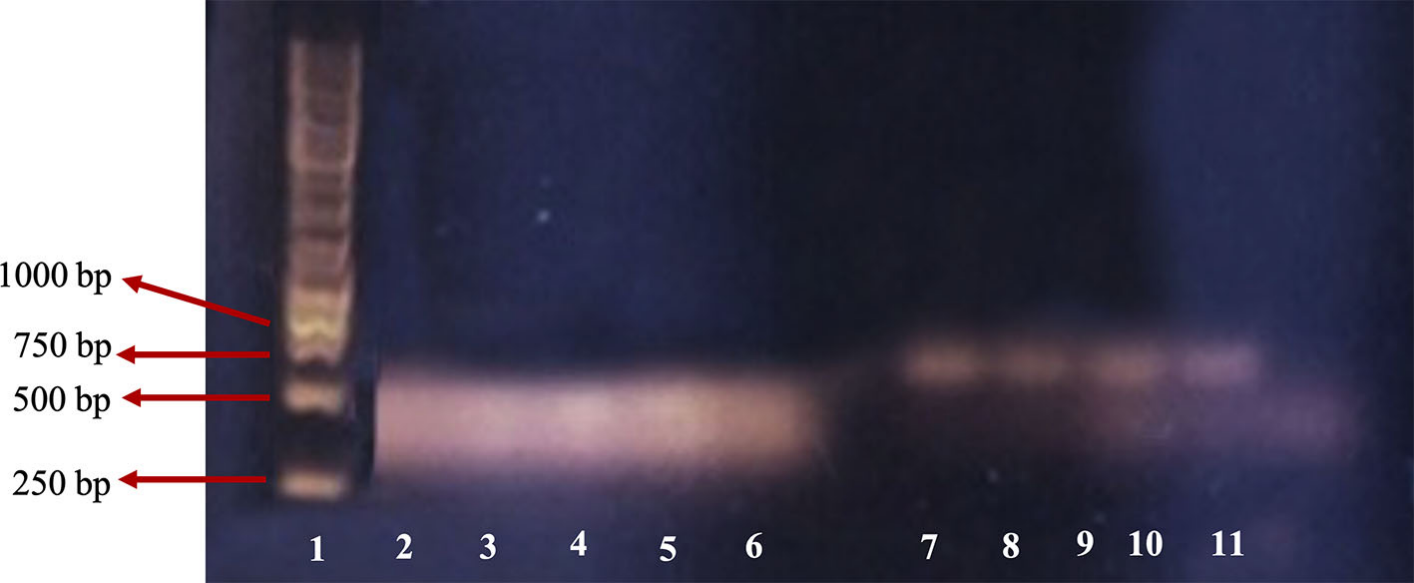
DNA PCR fragment of β-actin and β-tubulin 3 on 1% agarose gel. 1: leaders; 2: β-actin of M4 group; 3: β-actin of M3 group; 4: β-actin of M2 group; 5: β-actin of M1 group; 6: β-actin of control; 7: β-tubulin 3 of M4 group; 8: β-tubulin 3 of M3 group; 9: β-tubulin 3 of M2 group; 10: β-tubulin 3 of M1 group; 11: β-tubulin 3 of negative control.

In this study, the existence of nerve cells was (β-tubulin 3) confirmed using the RT-PCR. The control primer used in this study was β-actin. The β-actin primer was used to detect the actin gene, which is one of the housekeeping genes and the gene that is expressed within the cells in any tissue at any development stage of nerve cells. ^26^ At the same time, β-tubulin 3 primer was used to confirm nerve cell gene expression. ^13^ Figure 3 shows the DNA RT-PCR product of β-actin and β-tubulin.

For the β-actin gene, PCR product was found in lanes two, three, four, five and six representing M4 (1.0 mg/mL), M3 (0.9 mg/mL), M2 (0.8 mg/mL), M1 (0.7 mg/mL), and the control group, respectively. DNA bands in lanes two, three, four and five are similar to those in the control (lane six). This indicated that RNA extraction was successful and no PCR inhibitor was found in the RNA solution extraction product.

For the β-tubulin 3 gene, there were positive DNA bands in lanes seven, eight, nine and 10 that represented M4 (1.0 mg/mL), M3 (0.9 mg/mL), M2 (0.8 mg/mL), and M1 group (0.7 mg/mL), respectively. Each band had clear and clean PCR product at the same size, while there was no PCR product in the negative control which suggests that the cells were nerve cells. The control band was not clear, perhaps due to a pipetting error. The size of the DNA band of the primer β-actin was about 443bp as expected, and the primer β-tubulin 3 was about 700bp, while according to Wang *et al*., ^11^ the size of targeted β-tubulin 3 was 55bp. This size difference was probably because the primer design was based on *Rattus novergicus*, the brown rat, while in this study we used *M. musculus.* Based on sequence data DNA similarity of the rat and mouse are high (90%), the primer also has potential for β-tubulin 3 detection in the mouse. However, confirmation of the PCR product with sequencing is required.

## Conclusions

The application of *C. odorata* extract containing flavonoids and alkaloids increased the differentiation of MSCs into nerve cells at the optimum concentration of 0.8 mg/ml. Identification with RT-PCR targeting β-actin and β-tubulin 3 confirmed the presence of nerve cell genes.

## Data availability

### Underlying data

Figshare: Underlying data for ‘The differentiation of mesenchymal bone marrow stem cell into nerve cell induced by *Chromolaena odorata* extracts’. https://doi.org/10.6084/m9.figshare.19126544. ^27^

This project contains the following underlying data:

• Master table.xlsx [Table containing the raw data of the study]
• PCR gel.jpg [Raw picture of the PCR gel]

### Reporting guidelines

Figshare: ARRIVE checklist for ‘The differentiation of mesenchymal bone marrow stem cell into nerve cell induced by *Chromolaena odorata* extracts’. https://doi.org/10.6084/m9.figshare.19126544. ^27^

Data are available under the terms of the Creative Commons Attribution 4.0 International license (CC-BY 4.0).
