# Supplementary material for: The differentiation of mesenchymal bone marrow stem cells into nerve cells induced by Chromolaena odorata extracts
Source: F1000Res. 2022 Jun 15;11:252. Originally published 2022 Mar 1. [Version 2] doi: 10.12688/f1000research.108741.2 (PMC9214272; doi:10.12688/f1000research.108741.2)
Supplement: Peer review history

# Peer review: Reviewer response for version 2

**DOI:** 10.5256/f1000research.135035.r140951

**Published:** 2022-06-21

**Authors:** Arief Boediono

**Affiliations:** 1 Department of Anatomy, Physiology, and Pharmacology, Faculty of Veterinary Medicine, IPB University, Bogor, Indonesia

The author has made revisions according to the comments given.

 I have no further comments to the article.

Is the work clearly and accurately presented and does it cite the current literature?

Yes

If applicable, is the statistical analysis and its interpretation appropriate?

Yes

Are all the source data underlying the results available to ensure full reproducibility?

Yes

Is the study design appropriate and is the work technically sound?

Yes

Are the conclusions drawn adequately supported by the results?

Partly

Are sufficient details of methods and analysis provided to allow replication by others?

Partly

Reviewer Expertise:

Cell culture, stem cell, embryo engineering

I confirm that I have read this submission and believe that I have an appropriate level of expertise to confirm that it is of an acceptable scientific standard.

# Peer review: Reviewer response for version 2

**DOI:** 10.5256/f1000research.135035.r140952

**Published:** 2022-06-20

**Authors:** Sharun Khan

**Affiliations:** 1 Division of Surgery, ICAR-Indian Veterinary Research Institute, Bareilly, Uttar Pradesh, India

The authors have considered my comments while revising the manuscript.

Is the work clearly and accurately presented and does it cite the current literature?

Yes

If applicable, is the statistical analysis and its interpretation appropriate?

Yes

Are all the source data underlying the results available to ensure full reproducibility?

Yes

Is the study design appropriate and is the work technically sound?

Yes

Are the conclusions drawn adequately supported by the results?

Yes

Are sufficient details of methods and analysis provided to allow replication by others?

Yes

Reviewer Expertise:

Regenerative medicine, Stem cell therapy, Animal models

I confirm that I have read this submission and believe that I have an appropriate level of expertise to confirm that it is of an acceptable scientific standard.

# Peer review: Reviewer response for version 1

**DOI:** 10.5256/f1000research.120159.r128658

**Published:** 2022-06-08

**Authors:** Arief Boediono

**Affiliations:** 1 Department of Anatomy, Physiology, and Pharmacology, Faculty of Veterinary Medicine, IPB University, Bogor, Indonesia

The manuscript reported the influence of *C. odorata* leaf methanol extract to induce the differentiation of bone marrow MSCs into nerve cells using an *in vitro* experiment. In general, the manuscript provided good information, but some revisions need to be done before it can be indexed.

1. The dose used must match the content of the chemicals used (gentamicin, ketamine, xylazine, etc).
2. Cell concentration: please use 10 ^6^ instead of 106.
3. Whether the method for enumeration of differentiation nerve cells is standard, needs to be given reference. Table 2, Cell numbers (Mean + SD) need to be explained per 16 fields of view.
4. Delete Figure 2, it is a repetition of the data in Table 2.
5. In the conclusion: remove the words ‘containing flavonoids and alkaloids’ because in this study there was no identification of these active substances.

Is the work clearly and accurately presented and does it cite the current literature?

Yes

If applicable, is the statistical analysis and its interpretation appropriate?

Yes

Are all the source data underlying the results available to ensure full reproducibility?

Yes

Is the study design appropriate and is the work technically sound?

Yes

Are the conclusions drawn adequately supported by the results?

Partly

Are sufficient details of methods and analysis provided to allow replication by others?

Partly

Reviewer Expertise:

Cell culture, stem cell, embryo engineering

I confirm that I have read this submission and believe that I have an appropriate level of expertise to confirm that it is of an acceptable scientific standard, however I have significant reservations, as outlined above.

# Response

**Published:** 2022-06-12

**Authors:** Kartini Eriani

**Affiliations:** Universitas Syiah Kuala, Indonesia

Thank you for reviewing our manuscript. We believe the comments and suggestions improve the quality of our current manuscript.

 1. We have provided the actual concentration of the drugs used in this study. We also have change the amount of ketamine and xylazine from ml to mg/kg within the **Housing and husbandry of animals subheading**. 

 2. We have corrected the concentration of the cells from 106 to 10 ^6^ cells within the subheading, **Mesenchymal stem cells exposure with *C. odorata* extract.**

 3. We have provided the reference for enumeration of differentiation nerve cells within the subheading, **Enumeration of differentiation nerve cells**. We have provided the note on Table 2 that the number of cell numbers and the means based on measurements in 16 fields of view with 100x magnification.

 4. We have deleted the Figure 2 since the data is repetitive with Table 2. 

 5. We have deleted the words of “containing flavonoids and alkaloids” within the heading Conclusion.

# Peer review: Reviewer response for version 1

**DOI:** 10.5256/f1000research.120159.r125785

**Published:** 2022-03-14

**Authors:** Sharun Khan

**Affiliations:** 1 Division of Surgery, ICAR-Indian Veterinary Research Institute, Bareilly, Uttar Pradesh, India

The authors have attempted to evaluate the potential of *C. odorata* leaf methanol extract to induce the differentiation of bone marrow MSCs into nerve cells using an *in vitro* experiment. The manuscript is well written, presented and discussed, and understandable to a specialist readership. The findings are promising and the authors can further conduct studies to identify the specific constituent(s) that contribute to the differentiation potential of *C. odorata* leaf methanol extract. In general, the organization and the structure of the article are satisfactory and in agreement with the journal instructions for authors.

**Minor comments:**

1. Mesenchymal stem cells exposure with *C. odorata* extract - please correct "106 cells" to "10 ^6^ cells".
2. 2The mice were anesthetized with 0.01 mL of ketamine (Troy Laboratories PTY Limited) and 0.01 mL of xylazine - please mention the dose in mg/kg.
3. The authors are advised to comment on whether they have characterized the isolated mice MSCs according to the minimal criteria set by the International Society for Cellular Therapy (ISCT) ^1^^,^^2^.

Is the work clearly and accurately presented and does it cite the current literature?

Yes

If applicable, is the statistical analysis and its interpretation appropriate?

Yes

Are all the source data underlying the results available to ensure full reproducibility?

Yes

Is the study design appropriate and is the work technically sound?

Yes

Are the conclusions drawn adequately supported by the results?

Yes

Are sufficient details of methods and analysis provided to allow replication by others?

Yes

Reviewer Expertise:

Regenerative medicine, Stem cell therapy, Animal models

I confirm that I have read this submission and believe that I have an appropriate level of expertise to confirm that it is of an acceptable scientific standard.

## References

[1] : Definition and Characteristics of Mesenchymal Stromal Cells in Preclinical and Clinical Studies: A Scoping Review. *Stem Cells Translational Medicine* .2022;11(1) : 10.1093/stcltm/szab009 44-54 10.1093/stcltm/szab009 PMC889549135641170

[2] : Undifferentiated mouse mesenchymal stem cells spontaneously express neural and stem cell markers Oct-4 and Rex-1. *Cytotherapy* .2006;8(3) : 10.1080/14653240600735875 228-42 10.1080/14653240600735875 16793732

# Response

**Published:** 2022-06-12

**Authors:** Kartini Eriani

**Affiliations:** Universitas Syiah Kuala, Indonesia

Thank you for reviewing our manuscript. We believe the comments and suggestions improve the quality of our current manuscript.

 1. We have corrected the concentration of the cells from 106 to 10 ^6^ cells within the subheading, **Mesenchymal stem cells exposure with *C. odorata* extract**.

 2. We have changed the amount of ketamine and xylazine from ml to mg/kg within the **Housing and husbandry of animals** subheading. 

 3. We have added the information that the characterization of isolated MSCs according to the criteria by the International Society for Cellular Therapy (ISCT). Can be found within the subheading, **Number of differentiated mesenchymal stem cells into nerve like cells**
